# *In vitro* anti-cancer activity of siddha metallo-mineral formulation from Thanga uram with Hela cell lines

**DOI:** 10.6026/9732063002001603

**Published:** 2024-11-30

**Authors:** Sathya Rathish Mohana Jeevanath, Manjari Venkatraman, Kalaivanan Karuppan, Abarna Balasubramani, Natarajan Mohan, Siva Annamalai

**Affiliations:** 1 Department of Nanju Maruthuvam, National Institute of Siddha, Dr.M.G.R Medical University, Chennai - 47, Tamil Nadu, India; 2 Department of Siddha Medicine, Government Primary Health Centre, Perungattur, Tiruvannamalai - 604402, Tamil Nadu, India; 3 Department of Clinical Research, Siddha Regional Research Institute (Central Council for Research in Siddha, Ministry of Ayush, Govt. of India) Kuyavarpalayam, Puducherry, India

**Keywords:** Anti-cancer, annexin V flowcytometry, HeLa cell line, MTT assay, siddha, thanga uram

## Abstract

The second most common malignant tumour in women worldwide, cervical cancer seriously jeopardizes their health. It has been established that a high-risk Human papillomavirus (HPV) infection is the cause of cervical cancer. Cervical cancer is associated with several risk factors, including reproductive, behavioural and sexual characteristics. Many Siddha practitioners often prescribe Thanga uram (TU), a Siddha metallo-mineral formulation, for genito-urinary diseases, infertility, varicose veins and leucorrhoea. This study aims to evaluate the anti-cancer activity of "Thanga uram" against HeLa cell lines. Hela cell lines were used to assess the antiproliferative activity TU using the MTT assay and apoptosis by Annexin V flow cytometry. Thanga uram produced dose-dependent cytotoxic effects against HeLa cell lines with an LC50 value of 145.891µg/mL. Further investigation, using Annexin V flow cytometry, equivalently revealed the late apoptotic effect, with a percentage value of 28.15. Thus, the current study evaluates the effectiveness of Thanga Uram against cervical cancer.

## Background:

Cervical cancer ranks fourth among cancer-related deaths in women worldwide. In 2020, 3, 42,000 women died of cervical cancer, while 6, 04,000 were diagnosed with the disease, according to GLOBOCAN [1]. Recurrence rates range from 10-20% for stages I and II of the International Federation of Gynaecology and Obstetrics (FIGO) and upto 50-70% for advanced cases [2]. The rapid growth of cities, changes in lifestyle and increased environmental pollution are contributing factors to the rising incidence of cervical cancer, with a more pronounced impact in areas undergoing swift industrial development [3]. The peak age of incidence for cervical cancer in India is 50-59 years, compared to 35-44 years in developed countries [4]. With cervical cancer cases on the rise in recent years, the need for groundbreaking treatment solutions has become even more critical. Although the current relative survival rate of 48.7% offers hope for those diagnosed, it also highlights the significant room for improvement. By exploring innovative treatment modalities, we can work towards enhancing patient outcomes, increasing survival rates and effectively addressing this escalating health issue [3]. While chemotherapy, radiation and surgery are common and effective treatments for cervical cancer, they can also have a substantial impact on a patient's quality of life due to their debilitating side effects [5]. Siddha medicine is a traditional system of medicine that promotes health and treats a wide range of chronic illness. In the ancient Siddha literature, cancer is referred to by various terms, including 'Puttru', 'Vippuruthu' and 'Kazhalai'. Specifically, cervical cancer is denoted as 'Yoni Puttru' or 'Karuppai Kazhunthu Kazhalai', which translates to 'uterine tumor' or 'dark-colored cervical growth', respectively. These terms reflect the traditional understanding and description of cancerous conditions in the Siddha system of medicine. Siddha may be very significant in oncology as a preventive, anti-cancer treatment and adjuvant to chemotherapy since it enhances the patient's quality of life and minimizes the impact of the illness. Several formulations are indicated for cancer treatment in Siddha literature and numerous *in-vitro* studies have evaluated the anti-cancer properties of some medicines. As mentioned in the text Siddha Formulary of India, Thanga Uram (TU) is a Siddha metallo-mineral medicine indicated for a variety of illness including genito urinary problems, leucorrhoea, cognitive enhancement [6- 7]. Thanga Uram (TU) has previously been the subject of numerous investigations to assess its pharmacological activity, including styptic, memory-enhancing and *In vivo* spermatogenic activity [8]. However, there is still lack of evidence to support Thanga uram, the test medication, having anti-proliferative properties. Thus, using the MTT Assay and Apoptosis - Annexin V flowcytometry, we examined the anti-cancer efficacy of Thanga uram (TU) against HeLa cell lines.

## Materials and Methods:

The test drug TU was procured from a GMP certified pharmaceuticals, Tamil Nadu. The study was done in Biogenix Research Centre laboratory in Trivandrum.

## Cell line:

At the National Centre for Cell Sciences (NCCS), Pune, India, the HeLa cells (Human cervical cancer) were initially procured and it was then maintained in Dulbecco's modified Eagles medium, DMEM.

## MTT Assay:

A 25 cm2 tissue culture flask containing DMEM supplemented with 10% FBS, L-glutamine, sodium bicarbonate (Merck, Germany) and an antibiotic solution containing 100 U/ml of penicillin, 100 µg/ml of streptomycin and 2.5 µg/ml of amphoteracin B was used to cultivate it. Cell lines that were cultured were maintained at 37°C in an NBS Eppendorf, Germany and humidified 5% CO2 incubator. The MTT assay method was used to assess the vitality of the cells after they were directly observed using an inverted phase contrast microscope. Using trypsinization, a confluent monolayer of cells that was two days old was suspended in 10% growth media. A 96-well tissue culture plate was seeded with 100µl of the cell suspension (5x103 cells/well), which was then incubated at 37°C in a humidified 5% CO2 incubator. Using a cyclomixer, 1 mg of TU was weighed and dissolved in 1ml of 0.1% DMSO. To guarantee sterility, the sample solution was filtered using a 0.22 µm Millipore syringe filter. The growth medium was removed after 24 hours and each freshly prepared test compound TU in DMEM was serially diluted five times by a two-fold dilution (100µg, 50µg, 25µg, 12.5µg, 6.25µg in 500µl of DMEM). Three duplicates of each concentration of 100µl were added to each well and the mixture was then incubated at 37°C in an incubator with 5% CO2 that was humidified. Control cells that had not been treated were also kept. Following a 24-hour treatment period, the entire plate was examined using an inverted phase contrast tissue culture microscope (Olympus CKX41 equipped with an Optika Pro5 CCD camera) and the microscopic observations were captured as photographs. Any discernible alterations in the cell's shape, such as rounding or contracting, granulation and vacuolization in the cytoplasm of the cells, were regarded as markers of cytotoxicity.

## Apoptosis by annexin V flow cytometry :

The HeLa cell line was cultivated using the conventional techniques previously mentioned. Once sufficient confluency was reached, LC50 concentration of the sample, 145.891µg/ml was added and the mixture was incubated for a full day. Additionally, untreated control wells were kept up. The sample of cells was moved into a polystyrene tube measuring 12 by 75 mm. For fixation in a tube, 1x106 cells are the bare minimum advised. After that, the samples were centrifuged for five minutes at 3000 RPM. The particle was not disturbed during the removal of the supernatant. The cell pellet either produces a visible pellet or a white film on the tube bottom following centrifugation. Add 100 µL of the Muse TM Annexin V & Dead Cell Reagent to each tube in the tubes. After fully mixing the tubes for three to five seconds with a medium-speed pipette or vortex, they were allowed to sit at room temperature in the dark for twenty minutes. Muse flow cytometry software was used to evaluate the cells in a flow cytometer. Using Muse FCS 3.0 software, cells were gated against untreated control cells and examined for apoptosis [9].

## Results:

The LC50 Value is the 50% of cell death at a specific concentration, it was calculated using ED50 PLUSV1.0 Software and found to be 145.891µg/mL. The percentage viability changed with varying sample concentration along the horizontal axis. Each experiment was carried out three times and the findings are shown as Mean +/- SE. Data analysis was done using the Dunnets test and one-way ANOVA ***p < 0.001 in contrast to the control group. The below Table 1 represents the results of MTT Assay of the sample TU at varying concentrations (Figures 1 and 2 - see PDF).

## Apoptosis profile:

The early apoptotic percentage was determined to be 1.25%, while the late apoptotic percentage was 4.05% (Figures 3 and 4 - see PDF). The amount of debris assessed was 6.60%, contributing to a total apoptotic percentage of 5.30 In the sample of treated cells, 1.35% was in early apoptosis, 26.80% were in late apoptosis, 13.70% constituted debris, resulting in a total apoptotic rate of 28.15%.

## Discussion:

The current methods of treatment for cervical cancer are radiation therapy, chemotherapy and surgery [10]. Although these treatments have helped several people, the high rates of metastasis and recurrence eventually resulted in death. They have many adverse effects, including sexual and urological dysfunction, which can seriously impair the quality of life [11, 12]. Finding alternative medications that are equally effective and have fewer side effects is important for managing cervical cancer. These days, conventional treatments and traditional medicines are the main focus of cancer research. An integrated approach to Conventional medicine is necessary to deal with this awful illness.

## The Siddha system of medicine is based on three fundamental principles:

Vali (movement), azhal (transformation) and iyam (structure). In the context of cancer, azhal plays a crucial role as a regulator of cellular digestion and metabolic activity. An imbalance between azhal and iyam (the relationship between fire and matter) contributes to uncontrolled tissue growth, a hallmark of cancer. When azhal is diminished, a metaphorical crisis ensues, leading to an overactivation of vali (movement) and iyam (structure) forces, which in turn promotes cellular proliferation. Furthermore, alterations in iyam compromise immune function and tumour integrity, while changes in vali facilitate tumour growth and metastasis. This understanding, rooted in Siddha principles, offers a unique perspective on the pathophysiology of cancer and potential avenues for therapeutic intervention. Several compound formulations have been indicated for cervical cancer and thanga uram is one among them [3, 9, 13]. The ingredients of Thanga uram, Rasam (Mercury), Gandhagam (Sulphur) [14], Navaacharam (Ammonii chloridum), Velvangam (Tin - Stannum) [15], phyto-chemicals and the extracts present on these ingredients as well as the formulations based on these ingredients such as Velvanga parpam [16], Rasagandhi mezhugu [17], Rasaparpam also revealed the potent anti-cancerous properties [18, 19] also revealed the poten anti-cancerous properties.

The MTT assay revealed a dose-dependent decrease in cell viability. The percentage of cell viability decreases with an increase in the concentration. In 6.25 it is found to be 98.35 whereas at 100, it was found to be 66.57. The LC50 value was found to be 145.891 µg/ml. The LC50 value represents the lethal concentration, at which 50 percent of the cells were inhibited on treatment with the test drug. Annexin V binding assays are a crucial tool for accurately measuring apoptosis and differentiating it from necrosis. This process involves the translocation of phosphatidylserine from the inner to the outer membrane leaflet, where it is bound by annexin V in a calcium-dependent manner. In contrast, propidium iodide can penetrate compromised cell membranes and intercalate into DNA, allowing for the identification of necrotic and late-stage apoptotic cells. By combining annexin V and propidium iodide, researchers can reliably detect and quantify apoptosis in cervical cancer cells. The technique was employed to assess the apoptotic response of cervical cancer cells to a treatment, with the LC50 value indicating the concentration at which 50% of cells undergo apoptosis. The extent of apoptosis was then comprehensively evaluated using annexin V flow cytometry, providing valuable insights into the cellular response to treatment and the efficacy of the therapeutic approach. The results of apoptosis Annexin V flow cytometry reveal that when compared to the control and drug-treated groups, the late apoptotic changes in the control group were found to be 4.05% whereas in the drug-treated group, it is found to be 26.80%. The debris and total apoptotic percentage in the control group were found to be 6.60 and 5.30%, whereas in the test drug-treated group it was found to be 13.70 and 28.15 % respectively. This shows a significant increase in cell death in the drug-treated group when compared to the control group, proving the apoptotic activity. From this, it is evident that the test drug TU possesses significant anti-cancer activity and by future studies such as *in-vitro*, *in-vivo* studies and clinical trials should be conducted to emphasize its potential in the treatment of cancer.

## Conclusion:

The drug showed a significant effect in the cytotoxicity decreasing the cell viability in dose-dependent manner and the late apoptosis percentage from the results of flow cytometry in the test drug treated group with LC50 value revealed their potency to induce apoptosis to a certain extent. Further *in-vivo* and clinical trials should be carried out to prove its therapeutic effect in the management of cervical cancer.

## Author's contribution:

All authors are contributed equally to this study.

## Figures and Tables

**Table 1:** TU's cytotoxic effects on cancer cells treated with different cell viability percentage concentrations

**Sample Concentration (µg/ml)**	**Percentage Viability**
**Control**	**100**
TU -6.25	98.35
TU -12.5	91.5
TU -25	83.12
TU -50	76.57
TU -100	66.57
